# Restoration of shoulder kinematics during active throwing after the Latarjet procedure in anterior shoulder instability: A dynamic radiostereometric study

**DOI:** 10.1002/jeo2.70930

**Published:** 2026-10-11

**Authors:** Josephine Olsen Kipp, Emil Toft Petersen, Maiken Stilling, Anna Zejden, Rikke Jellesen Åberg, Thomas Falstie‐Jensen, Theis Muncholm Thillemann

**Affiliations:** ^1^ AutoRSA Research Group, Orthopaedic Research Unit Aarhus University Hospital Aarhus Denmark; ^2^ Department of Orthopaedic Surgery Aarhus University Hospital Aarhus Denmark; ^3^ Department of Clinical Medicine Aarhus University Aarhus Denmark; ^4^ Department of Radiology Aarhus University Hospital Aarhus Denmark

**Keywords:** anterior shoulder instability, biomechanics, Latarjet procedure, RSA, throw

## Abstract

**Purpose:**

To evaluate glenohumeral joint (GHJ) kinematics during a throwing motion in patients with anterior shoulder instability before and 1 year after the Latarjet procedure.

**Methods:**

In this prospective study, 13 patients with unilateral, traumatic anterior shoulder instability scheduled for the Latarjet procedure were examined preoperatively and at 1‐year follow‐up. GHJ kinematics were assessed during a standardized throwing motion using dynamic radiostereometric analysis. Preoperatively, both the injured and contralateral healthy shoulders were assessed. One year post‐operatively, only the injured shoulder was assessed. GHJ kinematics were evaluated in six degrees of freedom, including rotations and translations of the humeral head centre and contact point, as well as the glenohumeral contact area, throughout the throwing motion.

**Results:**

Post‐operatively, the humeral head centre shifted up to 1.1 mm posteriorly (95% confidence interval [CI]: 0.3–1.9) and 0.4 mm superiorly (95% CI: 0.0–0.7) compared with the injured shoulder and was 1.4 mm (95% CI: −1.2 to 4.1) more posterior compared with the healthy shoulder. The contact point translations showed similar patterns, although not statistically significant. The GHJ contact area increased post‐operatively by up to 87.0 mm^2^ (95% CI: 35.9–138.0) compared with the injured shoulder and with no statistically significant difference compared with the healthy shoulder.

**Conclusions:**

The Latarjet procedure produced a posterior–superior shift of the humeral head compared with the preoperative injured shoulder and restored glenohumeral contact area to levels similar to the healthy shoulder during an active throwing motion. These findings support the stabilizing effect of Latarjet procedures under functional, load‐bearing conditions relevant to overhead athletes.

**Level of Evidence:**

Level III.

**Abbreviations**
95% CI: 95% confidence intervals
CT: computed tomography
GHJ: glenohumeral joint
IQR: interquartile range
MCID: minimal clinically important difference
RSA: radiostereometry
SPM: statistical parametric mapping
WOSI: Western Ontario Shoulder Instability index

## INTRODUCTION

Anterior shoulder instability is common among young and athletic individuals, particularly in sports involving collision or overhead throwing motions [7, 20]. Surgical stabilization is often required, and the Latarjet procedure has become the preferred treatment in patients with glenoid bone loss or high athletic demands, as it is associated with lower recurrence rates, high patient satisfaction and faster return to sport compared with the Bankart repair [9].

Although clinical outcomes after the Latarjet procedure are well documented, its stabilizing effects on the glenohumeral joint (GHJ) during motion in vivo remain unknown. Most existing biomechanical studies have investigated static end‐range positions, which do not replicate the dynamic loads encountered during functional or sport‐specific activities [4, 18, 21]. The throwing motion is of particular interest, as the arm in the acceleration phase is positioned in abduction and external rotation, thereby exposing the humeral head to substantial anteriorly directed forces that challenge GHJ stability [5, 6]. Understanding these patterns is essential for clarifying the mechanisms underlying instability and for determining how surgical reconstruction restores GHJ stability under clinically relevant loading conditions and motions. However, dynamic in vivo GHJ kinematics during throwing‐specific motion after the Latarjet procedure have not previously been investigated.

GHJ kinematics during phases of the throwing motion have previously been investigated using optical tracking with bone pins or skin‐marker systems in healthy subjects [3, 13]. While bone pins provide accurate data, they are invasive, and skin markers are affected by motion artefacts and limited precision. Radiostereometric analysis (RSA) is a non‐invasive, high‐precision imaging technique that addresses these limitations and enables dynamic in vivo evaluation of GHJ kinematics [1, 2, 19].

The aim of this study was to evaluate GHJ kinematics during the acceleration phase of a throwing motion in patients with anterior shoulder instability, both before and 1 year after stabilization with the Latarjet procedure. We hypothesized that the Latarjet procedure restores GHJ stability, resulting in humeral head translations and glenohumeral contact area comparable to the healthy shoulder during an active throwing motion.

## MATERIALS AND METHODS

### Study design and participants

In this prospective cohort study, 20 patients with unilateral traumatic anterior shoulder instability (minimum two dislocations) were enroled at Aarhus University Hospital, Aarhus, Denmark, between February 2022 and March 2023. Inclusion criteria were age 18–40 years, a healthy contralateral shoulder, and more than 10% glenoid bone loss on computed tomography by best fit circle method (CT) [12]. Exclusion criteria were bilateral or multidirectional instability and alcohol or substance abuse. This study extends previous investigations of this cohort by evaluating dynamic GHJ kinematics during an active throwing motion, representing a functional, load‐bearing movement relevant to overhead activities [17, 18].

### Surgical procedure and test setup

All patients underwent the open Latarjet procedure performed by experienced shoulder surgeons (Level IV, according to the surgical skill classification system by Tang), according to the technique described by Young et al. [26, 28]. Approximately 25 mm of the coracoid process, including the conjoined tendon, was osteotomized and secured to the anteroinferior glenoid rim using two 4.5‐mm cannulated screws (Arthrex), and the capsule was reattached to the coracoacromial ligament stump.

Dynamic RSA (Adora RSA system, NRT X‐ray) combined with CT‐derived bone models (SOMATOM Definition Flash, Siemens Healthcare) was used to evaluate GHJ kinematics in six degrees of freedom both preoperatively and 1 year post‐operatively [25]. Preoperatively, both shoulders were examined; post‐operatively, only the operated shoulder was assessed. The contralateral shoulder was used as an internal control to minimize the influence of inter‐individual differences in GHJ anatomy and kinematics [24].

Kinematics were recorded during the acceleration and deceleration phases of a throwing motion, performed as an explosive movement from maximal external rotation to maximal internal rotation (Figure 1) [6]. The motion was performed against elastic resistance (TheraBand), starting with the elastic band in its fully extended position without initial tension. Resistance was individually adjusted (green, blue, black and silver) to provide maximal tolerable load, defined as the highest load at which the patient was able to perform a full throwing motion on the injured or post‐operative shoulder, with the contralateral shoulder loaded equally to the injured side.

**Figure 1:**
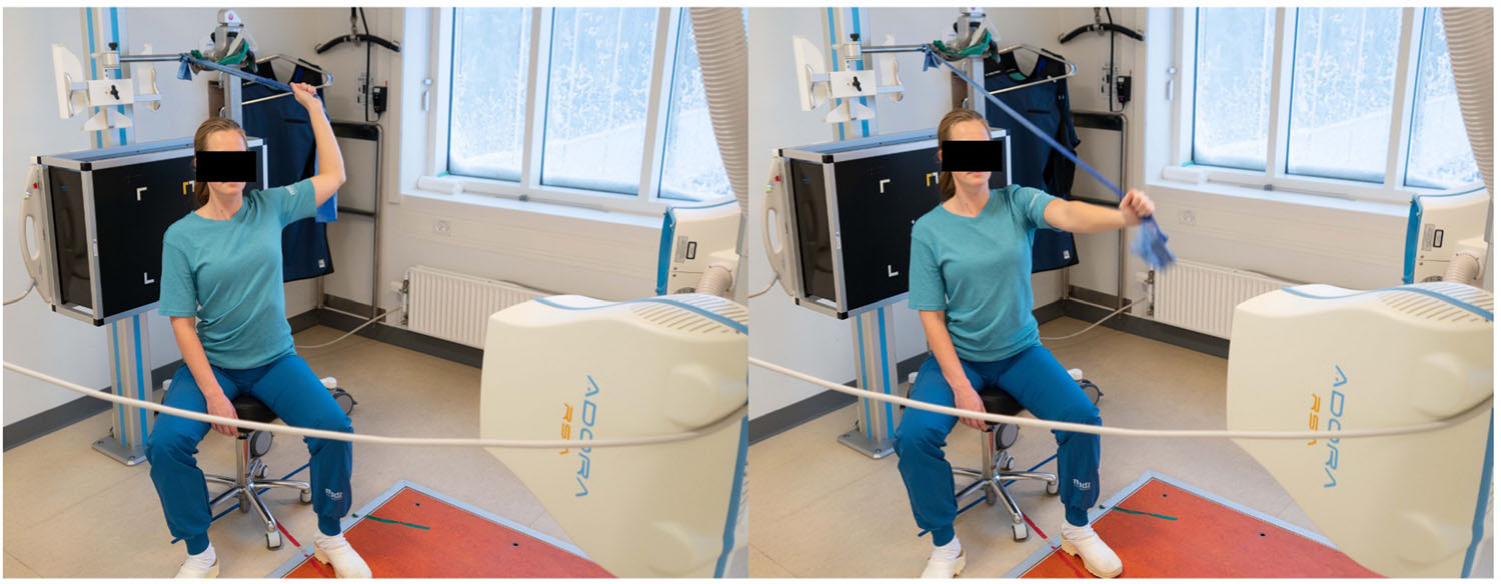
Test setup for the throwing motion recorded with radiostereometric analysis. The system consisted of two x‐ray tubes and detectors embedded within the calibration box. The motion was performed from maximal external rotation with the arm abducted and extended to maximal internal rotation.

### Data processing

CT‐derived bone models were registered to RSA recordings using AutoRSA software (Orthopedic Research Unit) as described by Olsen Kipp et al. [17]. GHJ kinematics was calculated in six degrees of freedom using Cardan angles (*ZXY*), with anatomical coordinate systems applied to the humerus and a glenoid‐oriented coordinate system to the scapula (Figure 2) [11, 27]. The scapular coordinate system was defined on the preoperative scapular bone model and subsequently transformed to the post‐operative bone model, ensuring a consistent reference frame across time points and enabling direct pre‐ to post‐operative comparisons. Three rotations: abduction(+)/adduction(−), flexion(+)/extension(−) and external rotation(+)/internal rotation(−) and two translations: anterior(+)/posterior(−) and superior(+)/inferior(−) were evaluated. The humeral head centre was defined as the origin of the humeral coordinate system relative to the glenoid‐oriented coordinate system. To evaluate the contact point and area, the distance between the humerus and glenoid was assessed within a range of 0–10 mm. The contact point was then calculated as the weighted proximity of the GHJ, while the contact area was defined as the region where the distance between the humeral head and glenoid was <6 mm, corresponding to the combined thickness of the glenoid and humeral cartilage [8, 29]. Motion data were normalized to a cycle from 0% to 100%, where 0% corresponded to maximal external rotation at the start of the acceleration phase and 100% to maximal internal rotation at the end of the deceleration phase.

**Figure 2:**
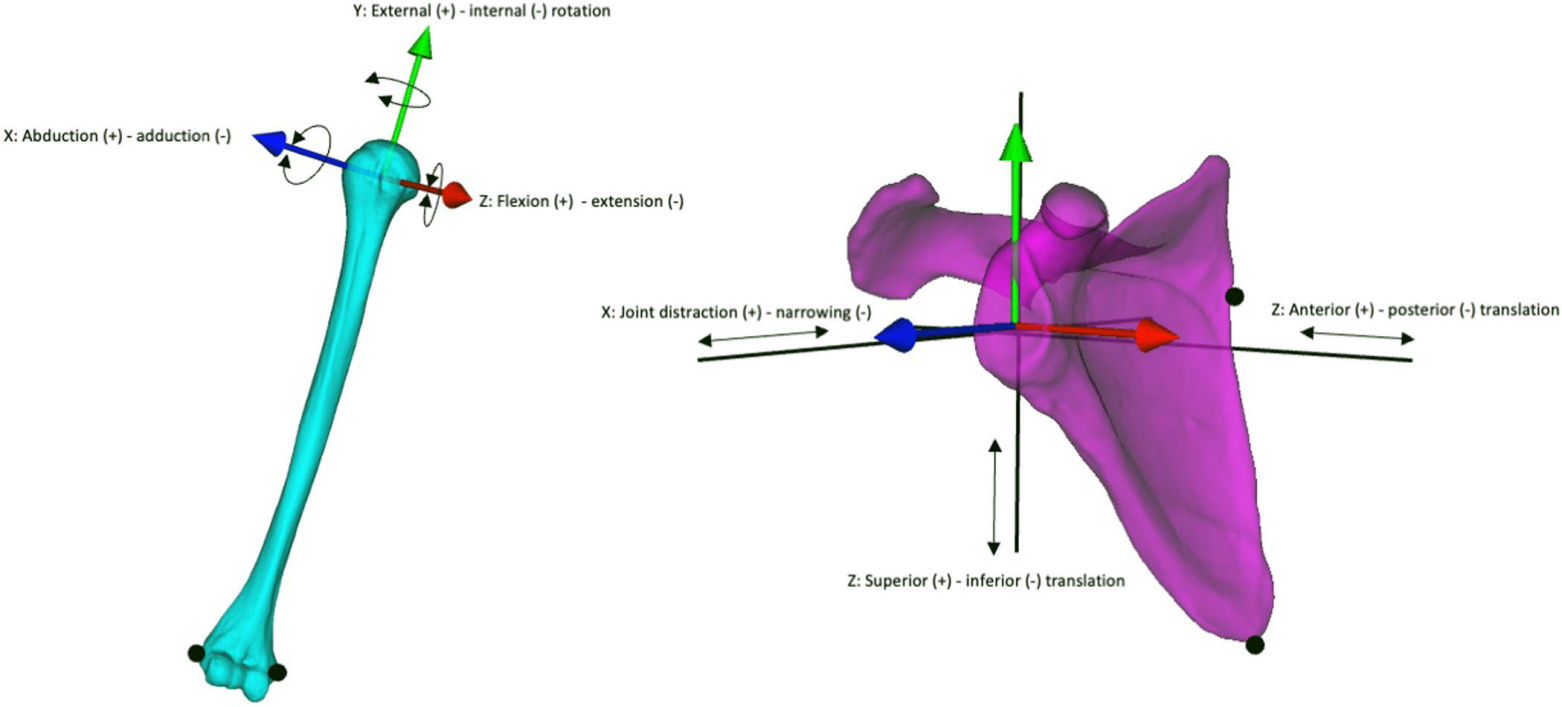
Anatomical coordinate system landmarks (black dots) for glenohumeral kinematic analysis. The humerus (cyan) and scapula (purple) are shown with orthogonal axes: *X*, *Y*, *Z*. Rotations of the humerus are defined as flexion(+)/extension(−), abduction(+)/adduction(−) and external(+)/internal(−) rotation. Humeral translations are defined relative to the scapular coordinate system as anterior(+)/posterior(−), distraction(+)/narrowing(−) and superior(+)/inferior(−).

### Statistics

Group comparisons across the motion trajectory were performed using a two‐sided paired Student's *t* test within the Statistical Parametric Mapping framework (SPM; spm1d.org, v.0.4.18, Python Software Foundation v.3.10), with a significance threshold of 0.05 [22]. Statistics were performed in accordance with the Checklist for Statistical Assessment of Medical Papers guidelines, and assumptions of randomness, homogeneity, and normality were verified [15]. For SPM analyses, both parametric and non‐parametric approaches were evaluated, with no differences observed between methods. Clinical outcomes and WOSI scores were assessed for normality using *Q*–*Q* plots. Normally distributed data were presented as mean with 95% confidence intervals (95% CIs). Non‐normally distributed data were presented as medians with interquartile ranges (IQRs) and compared using the Wilcoxon signed‐rank test.

### Clinical evaluation and patient‐reported outcomes

Pre‐ and post‐operatively, patients underwent a clinical examination, including range of motion measured with a goniometer and shoulder strength assessed as an isometric abduction with a spring scale (0–20 kg) in the 90° abducted and internally rotated position. Pre‐ and post‐operatively, patients completed the Western Ontario Shoulder Instability Index (WOSI), which was converted into a percentage score (0% indicating no symptoms). The minimal clinically important difference (MCID) is 220 points, corresponding to 10.4% [10].

### Sample size calculation

Sample size estimation was based on the study by Di Giacomo et al., which compared humeral head translation after the Latarjet procedure with that of healthy controls [4]. Due to limited comparable kinematic data after the Latarjet procedure, this was considered the best available reference despite differences in study design, including static MRI‐based assessment and the use of healthy controls rather than contralateral shoulders. Assuming a mean difference of 2.7 mm (standard deviations: 1.97 and 1.84), 12 patients were required to achieve 90% power at a 5% significance level.

## RESULTS

Twenty patients were included; data from 14 (13 male and 1 female) were obtained, as technical issues during dynamic RSA acquisition and data processing resulted in incomplete analyzable trajectories in the remaining recordings. The mean was 30 years (95% CI: 26–34). The mean glenoid bone loss was 17.6% (95% CI: 14.2–21.0). Six patients had injuries to the dominant arm, and 10 patients were sports‐active. Thirteen patients completed the 1‐year follow‐up, with one patient lost to follow‐up.

### Rotations during the throwing motion

During the throwing motion, GHJ rotational positions were assessed (Table S1). At the start of the throwing motion, no statistically significant differences were observed in abduction, flexion or external rotation position. At the end of the throwing motion, the GHJ of the post‐operative shoulder was 12.9° (95% CI: 4.3–21.4) less abducted compared with the injured shoulder, and 19.8° (95% CI: 11.1–28.6) less abducted compared with the healthy shoulder. Furthermore, at the end phase, the post‐operative shoulder was 8.4° (95% CI: 2.2–14.5) more flexed than the healthy shoulder. See Figure S1 for GHJ rotational kinematic trajectory during the throwing motion.

### Translations during the throwing motion

The kinematic results are presented as mean maximum translation range (95% CI) and mean maximal translation difference (95% CI) along the throwing motion trajectory. GHJ kinematics across the entire motion trajectory are presented in Figure 3, while values at the start, end and the minimum and maximum differences are summarized in Table 1.

**Figure 3:**
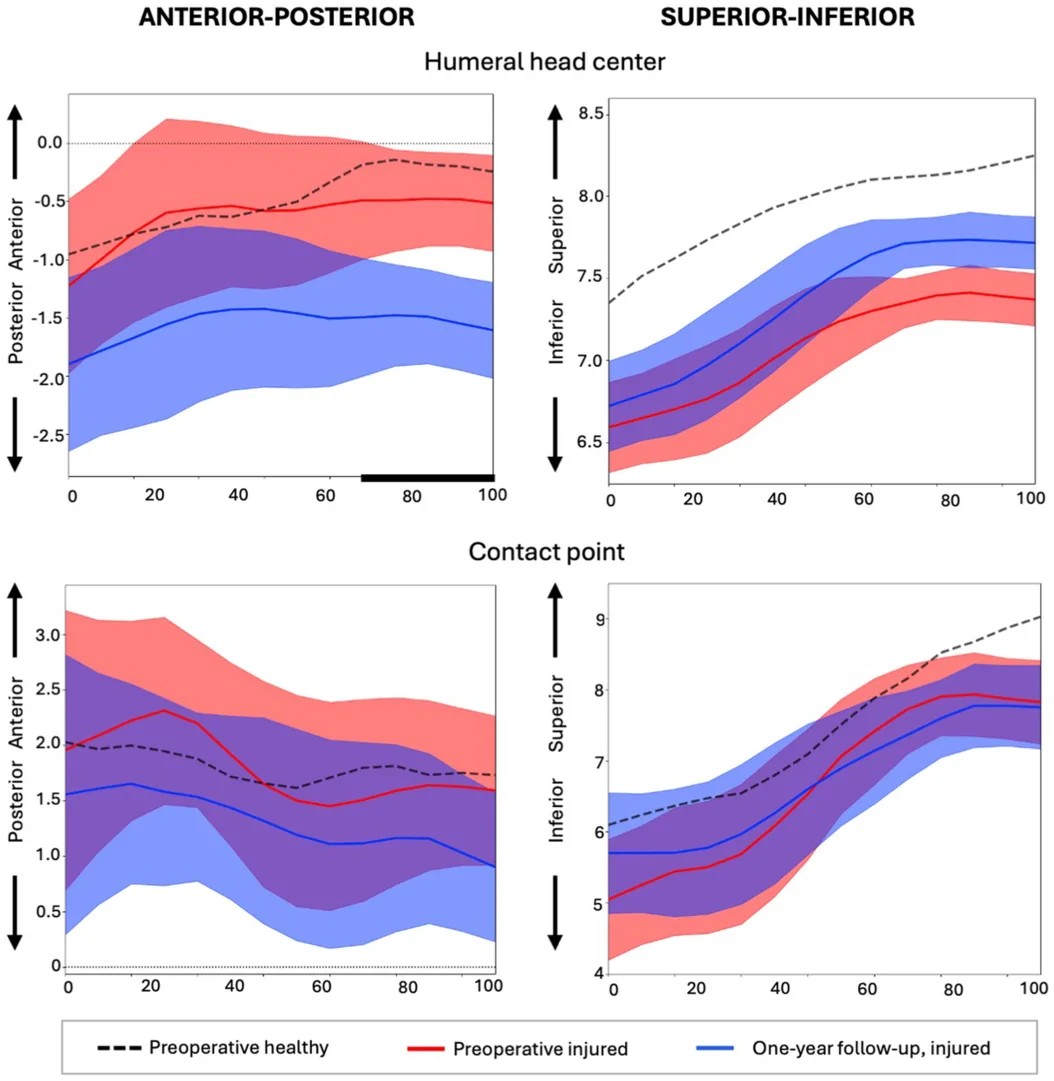
Humeral head centre and contact point translations (mm) during the throwing motion. Point 0 corresponds to the start of the throwing motion (start of the acceleration phase) and 100 to its end (end of the deceleration phase). The injured shoulder is shown in red, the post‐operative shoulder in blue (shaded areas: 95% CI), and the healthy shoulder as a black dotted line. Statistically significant differences between the injured and post‐operative shoulders are indicated by a black bar at the bottom. CI, confidence interval.

**Table 1:** Translations of the humeral head centre and contact point during the throwing motion.

	Start	End	Min	Max
Healthy minus injured (mm)				
AP: Humeral head centre	0.1 (−2.4 to 2.5)	−0.3 (−2.6 to 2.0)	−0.1 (−2.3 to 2.1) at 60%	0.4 (−2.0 to 2.8) at 20%
SI: Humeral head centre	−0.9 (−2.6 to 0.9)	−0.9 (−2.5 to 0.8)	−0.8 (−2.4 to 0.8) at 80%	−1.1 (−2.9 to 0.8) at 20%
AP: Contact point	0.2 (−1.6 to 2.1)	−0.1 (−1.5 to 1.3)	−0.0 (−1.6 to 1.6) at 80%	0.6 (−1.6 to 2.7) at 20%
SI: Contact point	−0.8 (−2.9 to 1.4)	−0.9 (−2.9 to 1.2)	−0.2 (−1.9 to 1.6) at 70%	−0.9 (−2.9 to 1.2) at 100%
Post‐operative minus injured (mm)				
AP: Humeral head centre	−0.7 (−2.2 to 0.8)	−1.1 (−1.9 to −0.3)	−0.7 (−2.1 to 0.8) at 70%	−1.1 (−1.9 to −0.3) at 100%*
SI: Humeral head centre	0.1 (−0.4 to 0.7)	0.3 (0.0–0.7)	0.1 (−0.4 to 0.7) at 70%	0.4 (0.0–0.7) at 60%*
AP: Contact point	−0.4 (−2.9 to 2.1)	−0.7 (−2.0 to 0.6)	−0.3 (−2.2 to 1.6) at 50%	−0.7 (−2.4 to 1.0) at 20%
SI: Contact point	0.7 (−1.0 to 2.3)	−0.1 (−1.2 to 1.1)	−0.1 (−1.2 to 1.1) at 90%	0.7 (−1.0 to 2.3) at 0%
Post‐operative minus healthy (mm)				
AP: Humeral head centre	−1.0 (−3.4 to 1.4)	−1.4 (−4.1 to 1.2)	−0.8 (−3.3 to 1.6) at 40%	−1.4 (−4.1 to 1.2) at 100%
SI: Humeral head centre	−0.7 (−2.9 to 1.6)	−0.6 (−2.4 to 1.3)	−0.4 (−2.2 to 1.3) at 80%	−0.8 (−3.1 to 1.5) at 20%
AP: Contact point	−0.4 (−3.1 to 2.4)	−0.7 (−2.6 to 1.2)	−0.1 (−2.2 to 1.9) at 10%	−0.7 (−2.6 to 1.2) at 100%
SI: Contact point	0.1 (−3.4 to 3.2)	−0.9 (−3.5 to 1.6)	0.0 (−3.4 to 3.2) at 10%	−0.9 (−3.5 to 1.6) at 100%

*Note*: Comparison of mean (95% confidence intervals) translations of the humeral head centre and contact point during the throwing motion between the healthy, injured, and post‐operative shoulders in the anterior–posterior (AP) and superior–inferior (SI) directions. Positive values correspond to anterior and superior translations. For the minimum and maximum differences, it is stated where in the throwing phase these occurred.

* Statistically significant differences.

During the throwing motion, the humeral head centre translated anteriorly and superiorly in all shoulders. Mean anterior translations were 2.1 mm (95% CI: 1.2–2.9) for the healthy shoulder, 1.7 mm (95% CI: 1.0–2.5) for the injured shoulder, and 1.7 mm (95% CI: 1.1–2.3) for the post‐operative shoulder. Mean superior translations were 1.3 mm (95% CI: 0.9–1.7), 1.0 mm (95% CI: 0.6–1.4) and 1.2 mm (95% CI: 0.8–1.7) for the healthy, injured and post‐operative shoulder, respectively. Comparing the humeral head centre translations from pre‐ to post‐operatively, the post‐operative shoulder demonstrated a more posterior position, with a mean maximal difference of 1.1 mm (95% CI: 0.3–1.9) than the injured shoulder, and 1.4 mm (95% CI: −1.2 to 4.1) than the healthy shoulder. The most pronounced differences occurred at the end of the throwing motion. In the superior–inferior direction, the post‐operative shoulder was positioned more superiorly throughout the motion, with a mean maximal difference of 0.4 mm (95% CI: 0.0–0.7) compared with the injured shoulder.

During the throwing motion, the contact point translated anteriorly and superiorly in all shoulders. Mean anterior translations were 2.0 mm (95% CI: 1.1–2.8) for the healthy shoulder, 2.0 mm (95% CI: 1.3–2.7) for the injured shoulder and 2.3 mm (95% CI: 1.5–3.1) for the post‐operative shoulder. Mean superior translations were 3.4 mm (95% CI: 2.3–4.5), 3.3 mm (95% CI: 2.1–4.6) and 3.0 mm (95% CI: 2.2–3.9), respectively. The contact point exhibited patterns similar to the humeral head centre translations; however, no statistically significant differences were observed among the healthy, injured and post‐operative shoulders.

### Contact area

The mean maximal GHJ contact area for the injured shoulder was 646.5 mm^2^ (95% CI: 614.5–678.3). Post‐operatively, the mean maximal contact area was consistently larger by up to 87.0 mm^2^ (95% CI: 35.9–138.0) compared with the injured shoulder. No statistically significant differences in contact area were found between the post‐operative and contralateral healthy shoulders, or between the injured and healthy shoulders, except at the beginning of the throwing motion, where the mean maximal contact area of the injured shoulder was up to 112.0 mm^2^ smaller (95% CI: 44.2–179.8) than that of the healthy shoulder. The GHJ contact areas are displayed in Figure 4 (mean maximal) and Figure 5 (illustration of the contact area on the glenoid of a representative patient).

**Figure 4:**
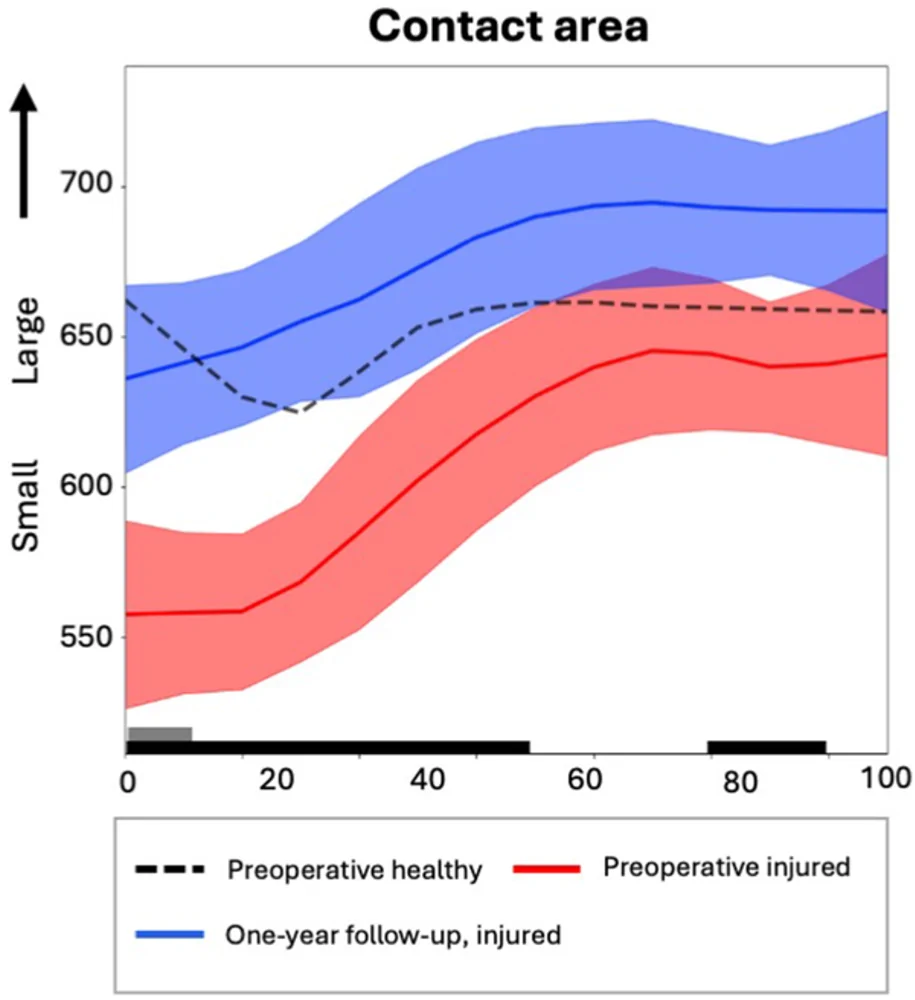
Contact area (mm^2^) during the throwing motion. Point 0 corresponds to the start of the throwing motion (start of the acceleration phase) and 100 to its end (end of the deceleration phase). The injured shoulder is shown in red, the post‐operative shoulder in blue (shaded areas: 95% CI), and the healthy shoulder as a black dotted line. Statistically significant differences are shown as bars at the bottom for injured versus post‐operative (black) and injured versus healthy (grey). CI, confidence interval.

**Figure 5:**
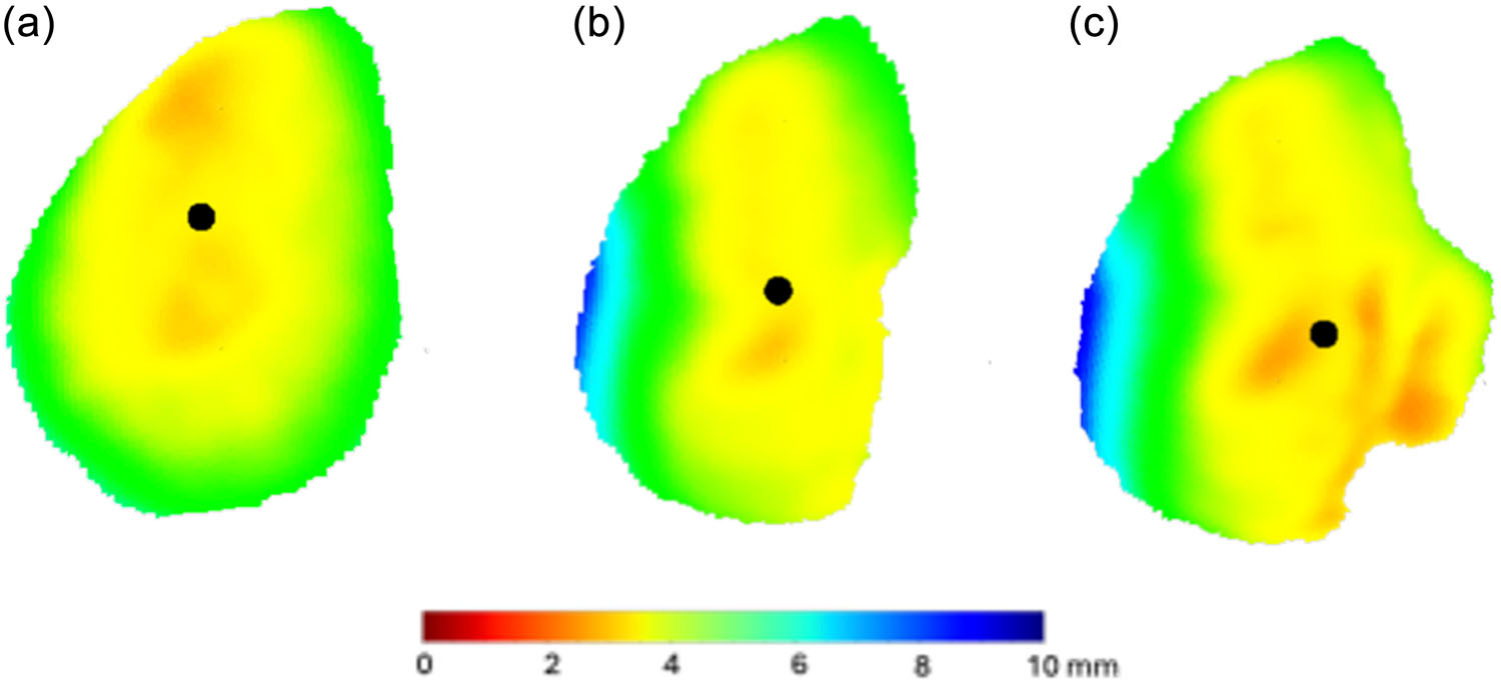
Illustration of the glenoid contact area in a representative patient for the healthy shoulder (a), injured shoulder (b) and post‐operative shoulder (c). The glenoids are displayed at the time point of the maximal contact area.

### Clinical outcomes

Clinical outcomes improved following surgery, with WOSI scores decreasing from 57.4% (95% CI: 50.5–64.4) preoperatively to 39.3% (95% CI: 23.2–55.4) post‐operatively. Shoulder strength and range of motion were generally comparable between the injured and post‐operative shoulders, although external rotation remained reduced compared with the healthy shoulder (Table S2).

## DISCUSSION

This study demonstrated that the Latarjet procedure resulted in a more posterior and superior humeral head position and increased glenohumeral contact area compared with the preoperative injured shoulder during an active throwing motion. To our knowledge, this is the first study to evaluate in vivo GHJ kinematics during a throwing‐specific motion following the Latarjet procedure.

### Stabilizing effect of the Latarjet procedure

Most studies on the stabilizing effect of the Latarjet procedure have examined passive or unloaded conditions, such as external rotation or the apprehension–relocation test [4, 18, 21]. Under these circumstances, no statistically significant differences from the healthy shoulder have been observed, suggesting that glenohumeral kinematics are restored. In contrast, one study examined GHJ kinematics of the active shoulder and demonstrated up to 2.7 mm of posterior translation following the Latarjet procedure compared with healthy shoulders [4]. In the present study, the post‐operative shoulder translated up to 1.6 mm posteriorly compared with the contralateral healthy side during an active throwing motion, although not statistically significant. These findings highlight the importance of assessing stability under clinically relevant, loaded conditions, as passive examinations may underestimate the functional effect of the procedure.

The clinical relevance of the observed differences in humeral head translation remains unclear, as no established threshold exists for clinically meaningful changes in glenohumeral kinematics measured by dynamic RSA [14]. Therefore, these findings should primarily be interpreted as biomechanical changes rather than a direct measure of clinical improvement. However, previous RSA studies have demonstrated that relatively small changes in humeral head position may occur following stabilization procedures [4, 18, 21]. In a study evaluating the apprehension position 1 year after the Latarjet procedure, the post‐operative humeral head position differed from the preoperative injured shoulder by 0.7 mm posteriorly and 0.8 mm superiorly, which was accompanied by resolution of apprehension symptoms [18]. Although testing conditions differ from the present study, these findings suggest that small but measurable changes in glenohumeral kinematics may contribute to improved perceived stability. Similarly, in the present study, the observed post‐operative shift towards a more centred humeral head position occurred alongside improved WOSI scores.

In addition to the magnitude of translation, the phase of the throwing motion in which these differences occurred may provide further insight into the stabilizing mechanisms of the Latarjet procedure. Notably, whereas previous studies found the greatest stabilizing effect with the arm abducted and externally rotated, the present study observed the largest differences at the end of the throwing phase, when the arm was adducted and internally rotated. This may partly reflect the resistance applied by the elastic band; however, the findings suggest that the combined effect of the coracoid bone block and the conjoined tendon sling contributes to stability throughout the entire throwing cycle, extending beyond positions previously described as most vulnerable to instability.

In the present study, differences in rotational glenohumeral kinematics were observed across test stages, particularly in abduction, where the operated shoulder consistently demonstrated reduced abduction compared with the other conditions. These differences occurred in the terminal phase of the motion, likely reflecting both the challenge of standardizing rapid, loaded movements and the mechanical restraints following surgery. These rotational differences may influence translational motion. Matsuki et al. examined the relationship between abduction angle and superior–inferior humeral head translation and reported a positive correlation between abduction and superior translation up to 105° [16]. In the present study, the operated shoulder was less abducted yet exhibited slightly higher superior humeral head positions, suggesting that positional differences do not account for this finding.

### Throw kinematics

Previous studies investigating GHJ kinematics during throwing motions have been limited by methodological constraints. Dal Maso et al., using bone pins in two healthy subjects, reported humeral head translations of up to 2.1 mm anteriorly and 3.6 mm superiorly during a ball throw [3]. While broadly consistent with the present findings of 2.1 mm anterior and 1.3 mm superior translation in healthy shoulders, the invasive nature of bone pins restricts their clinical application. Lädermann et al. employed a non‐invasive approach using skin markers to study tennis players with rotator cuff or labral lesions during flat and kick serves [13]. In the deceleration phase, they reported anterior translations of 8%–57% and superior translations of 5%–34% of the humeral radius, corresponding to approximately 1.2–8.3 and 0.9–4.9 mm, assuming a glenoid width of 29 mm [23]. Despite the application of global optimization to reduce tissue artefacts, the reported measurement accuracy of 1.9–3.3 mm introduces considerable variability, limiting the reliability of small‐joint translation measurements. In contrast, the method applied in the present study offers both high precision and non‐invasive assessment, enabling repeated kinematic measurements during dynamic activities such as throwing. The relatively small anterior and superior translations observed in the healthy shoulder highlight the subtle nature of GHJ motion and underscore the necessity of accurate measurement techniques to detect clinically relevant changes, particularly when evaluating the effects of surgical interventions or rehabilitation.

### Strengths and limitations

A key strength of this study is the use of dynamic RSA, which enables non‐invasive, highly precise three‐dimensional tracking of glenohumeral kinematics, enabling direct comparisons before and after surgery. Of the 20 included patients, only 14 had analyzable preoperative recordings from both shoulders. The main limitation of this study was that only selected phases of the throwing motion were performed; however, this ensured a more comparable range of motion. Furthermore, the throwing motion was performed with a Theraband rather than a ball, which may have introduced additional resistance during the terminal phase of the throw and resulted in different loading conditions between assessments, as resistance was individually adjusted to each patient's maximal tolerable load. Using a frame rate of only 10 Hz may have resulted in fewer captured points during fast movements, potentially affecting the fidelity of the interpolated data. Nevertheless, this approach ensured standardization and reproducibility, permitting valid within‐subject comparisons. Another limitation is the absence of cartilage in the bone model, which may influence contact area estimates; however, as cartilage distribution is generally homogeneous across the humerus and glenoid, this effect is likely minimal.

## CONCLUSION

This study presents dynamic RSA as a precise and non‐invasive method for evaluating in vivo GHJ kinematics during a throwing motion. The Latarjet procedure provided a more posterior and superior humeral head centre and increased the contact area similar to the healthy shoulder during the acceleration and deceleration phases of the throwing motion, supporting its role in maintaining joint stability under the high anterior forces encountered in overhead athletic activities.

## AUTHOR CONTRIBUTIONS

Josephine Olsen Kipp, Emil Toft Petersen, Theis Muncholm Thillemann and Maiken Stilling contributed to study conceptualization and design. Data collection was performed by Josephine Olsen Kipp, Anna Zejden, Rikke Jellesen Åberg, Thomas Falstie‐Jensen, Theis Muncholm Thillemann and M Stilling. Data curation and formal analysis were carried out by Josephine Olsen Kipp and Emil Toft Petersen. Josephine Olsen Kipp drafted the original manuscript, and all authors contributed to the revision, read and approved the final manuscript.

## CONFLICT OF INTEREST STATEMENT

The authors declare no conflicts of interest.

## ETHICS STATEMENT

Ethical approval was obtained from the Central Denmark Region Committees on Health Research Ethics (journal no. 1‐10‐72‐298‐21) and the Danish Data Protection Agency (journal no. 1‐16‐02‐344‐21). Written informed consent was obtained from all participants prior to participation.

## Supporting information

Supporting information.

## Data Availability

The data that support the findings of this study are available from the corresponding author upon reasonable request.
