# Supplementary material for: Restoration of shoulder kinematics during active throwing after the Latarjet procedure in anterior shoulder instability: A dynamic radiostereometric study
Source: J Exp Orthop. 2026 Oct 11;13(4):e70930. doi: 10.1002/jeo2.70930 (PMC13654321; doi:10.1002/jeo2.70930)
Supplement: Supplementary file 1 [file JEO2-13-e70930-s001.docx]

**Supplementary material**

**Restoration of shoulder kinematics during active throwing after the Latarjet procedure in anterior shoulder instability: A dynamic radiostereometric study**

*Josephine Olsen Kipp^1,3^, Emil Toft Petersen^1,2,3^, Maiken Stilling^1,2,3^, Anna Zejden^4^, Rikke Jellesen Åberg^4^, Thomas Falstie-Jensen^3^, Theis Muncholm Thillemann^2,3^*

^1^ AutoRSA Research Group, Orthopaedic Research Unit, Aarhus University Hospital, Aarhus, Denmark

^2^ Department of Clinical Medicine, Aarhus University, Aarhus, Denmark

^3^ Department of Orthopaedic Surgery, Aarhus University Hospital, Aarhus, Denmark

^4^ Department of Radiology Aarhus University Hospital, Aarhus, Denmark

**Figure S1** Rotational range of motion during the throwing motion. Time point 0% marks the start of the acceleration phase and 100% the end of the deceleration phase. The mean degrees for the injured shoulder is shown in red, for the postoperative shoulder in blue (shaded areas: 95% confidence intervals), and for the healthy shoulder in a black dotted line. Statistically significant differences are indicated by colored bars at the bottom: grey for healthy versus injured shoulders, black for injured versus postoperative shoulders, and green for healthy versus postoperative shoulders.

**Table S1** Rotation of the humeral head (mean degrees, 95%CI) during the throwing motion

|  | External rotation | Flexion | Abduction |
| --- | --- | --- | --- |
| Healthy **(°)**  ***Start***  ***End*** | 79.0 (73.3; 84.6)  40.0 (34.6; 45.4) | -24.7 (-31.0; -18.4)  -4.7 (-7.8; -1.6)^*^ | 64.9 (57.9; 72.0)  93.8 (89.4; 98.2) ^*#^*^!^* |
| Injured **(°)**  ***Start***  ***End*** | 71.0 (65.8; 76.3)  43.0 (37.4; 48.5) | -14.6 (-20.1; -9.1)  -0.0 (-5.2; 5.2)^*^ | 67.1 (61.4; 72.9)  85.6 (83.1; 88.1) ^*#^*^!^* |
| Postoperative **(°)**  ***Start***  ***End*** | 69.2 (62.8; 75.7)  37.2 (31.1; 43.3) | -16.2 (-23.7; -8.8)  3.5 (0.5; 6.6)^*^ | 64.2 (56.9; 71.4)  74.9 (70.0; 79.8) |

*Mean rotation angle with 95% confidence intervals. Positive values correspond to abduction, flexion, and external rotation. Statistically significant differences are indicated as follows: ^*^Healthy vs. postoperative shoulder; ^#^Healthy vs. injured shoulder (preoperative); ^!^Injured vs. postoperative shoulder.*

**Table S2** Clinical and patient-reported outcomes

| Outcome | Healthy | Injured | Postoperative |
| --- | --- | --- | --- |
| ****WOSI score (%)** *mean (95%CI)*** | – | 57.4% (50.5; 64.4)^*^ | 39.3% (23.2; 55.4)^*^ |
| ****Shoulder strength (kg)** *mean (95% CI)*** | 10.5 (8.7; 12.3)^#^ | 9.2 (7.4; 11.0)^#^ | 9.5 (6.7; 12.2) |
| ****Flexion (°)** *median (IQR)*** | 180 (170; 180) | 180 (170; 180) | 180 (170; 180) |
| ****Extension (°)** *median (IQR)*** | 50 (40; 60) | 40 (40; 50) | 50 (40; 60) |
| ****Abduction (°)** *median (IQR)*** | 170 (170; 180) | 170 (160; 180) | 180 (170; 180) |
| ****External rotation (°)** *median (IQR)*** | 70 (50; 80) ^#*^ | 50 (45; 80) ^#^ | 50 (35; 60)^*^ |

*WOSI: Western Ontario Shoulder Instability Index (lower score = better function), 95%CI: 95% Confidence Intervals; IQR: Interquartile range. Statistically significant differences are indicated as follows: ^*^Healthy vs. postoperative shoulder; ^#^Healthy vs. injured shoulder; ^!^Injured vs. postoperative shoulder.*
